# Human-Centric Digital Twins in Industry: A Comprehensive Review of Enabling Technologies and Implementation Strategies

**DOI:** 10.3390/s23083938

**Published:** 2023-04-12

**Authors:** Usman Asad, Madeeha Khan, Azfar Khalid, Waqas Akbar Lughmani

**Affiliations:** 1 Department of Mechanical Engineering, Capital University of Science and Technology, Islamabad 45750, Pakistan; 2 Department of Mechatronics Engineering, National University of Sciences and Technology, Islamabad 44000, Pakistan; 3 Digital Innovation Research Group, Department of Engineering, School of Science & Technology, Nottingham Trent University, Nottingham NG11 8NS, UK

**Keywords:** Digital Twin, human-centric, Industry 5.0, literature review, human-robot collaboration, artificial intelligence

## Abstract

The last decade saw the emergence of highly autonomous, flexible, re-configurable Cyber-Physical Systems. Research in this domain has been enhanced by the use of high-fidelity simulations, including Digital Twins, which are virtual representations connected to real assets. Digital Twins have been used for process supervision, prediction, or interaction with physical assets. Interaction with Digital Twins is enhanced by Virtual Reality and Augmented Reality, and Industry 5.0-focused research is evolving with the involvement of the human aspect in Digital Twins. This paper aims to review recent research on Human-Centric Digital Twins (HCDTs) and their enabling technologies. A systematic literature review is performed using the VOSviewer keyword mapping technique. Current technologies such as motion sensors, biological sensors, computational intelligence, simulation, and visualization tools are studied for the development of HCDTs in promising application areas. Domain-specific frameworks and guidelines are formed for different HCDT applications that highlight the workflow and desired outcomes, such as the training of AI models, the optimization of ergonomics, the security policy, task allocation, etc. A guideline and comparative analysis for the effective development of HCDTs are created based on the criteria of Machine Learning requirements, sensors, interfaces, and Human Digital Twin inputs.

## 1. Introduction

Industry has a constant need to evolve, innovate, and adopt technological advances in order to meet the challenges of the future in a competitive environment. Broadly in literature, historical industrial progress is divided into epochs called “Industrial Revolutions”, characterized by major technological paradigm shifts [1]. In light of the digital revolution and exponential growth in computational power, information and data processing capabilities, smart sensors, and computational intelligence, the focus of Industry 4.0 is to leverage emerging technologies to interconnect smart Cyber-Physical Systems (CPS) to enable mass customization in robust, flexible Smart Factories. A synergistic paradigm of emerging technologies is recently developing, which includes leveraging multi-physics simulations to create Digital Twins (DT) of physical systems. DTs use immersive technologies such as Virtual Reality (VR) and Augmented Reality (AR) to explore and interact with virtual objects and use collaborative robots for safe, intuitive Human-Robot Interaction (HRI), especially by utilizing advances in artificial intelligence. There is an increasing realization that in addition to technological aspirations, government, and industry should move towards a value-driven future where the focus is on human well-being, sustainability, and resilience under Industry 5.0 [2].

### 1.1. Cyber-Physical Systems and Digital Twins

In the context of Industry 4.0, certain enabling technologies such as distributed computing, sensor networks, big data, and the Internet of Things (IoT) have found increasing application in manufacturing settings to achieve reliability, flexibility, increased automation, and better performance [3]. Due to advances in computational intelligence, increasing data bandwidth and processing capability, and widespread use of sensors, it has become possible to create highly autonomous virtual replicas of CPS with advanced decision-making capabilities. These highly autonomous virtual replicas are known as Digital Twins. The term ‘Digital Twin’ is popularized by NASA in the context of flying vehicles [4]. Many definitions of DTs have been proposed in the literature. The definition proposed by AIAA [5] is “a set of virtual information constructs that mimic the structure, context, and behavior of an individual/unique physical asset, or a group of physical assets, are dynamically updated with data from their physical twins throughout their life cycles and inform decisions that realize value”.

Although the concept of DTs originated in the aerospace industry, they can now be used for the digital representation of any complex system [6]. DTs have been employed in many traditional manufacturing areas, including metallurgy, machining, grinding, and hole punching [7]. Functionally, DTs are used for supervision, interaction, and prediction of assets, often exploiting artificial intelligence and immersive user experiences, for example using Extended Reality (XR) [8]. Furthermore, Digital Twin technology is also finding increasing use by industry leaders, for example in the power production sector by British Petroleum for monitoring oil and gas facilities and General Electric for monitoring turbines [9].

This increasing interest of investigators in digitization originated a number of opportunities to explore the emerging phenomena of DTs for CPS to meet the requirements of smart manufacturing. Computer simulations with Multi-physics modeling can be employed to create DTs of real machines with a bi-directional data flow between the physical and virtual systems. Such DTs can be utilized for rapid testing, optimization, and deployment for flexible manufacturing using Cyber-Physical Production Systems.

### 1.2. Human Robot Collaboration

Industrial robots driven by Programmable Logical Controllers executing fixed sets of instructions have become a staple of automation and mass production in advanced factories. Most such industrial robots are entirely separated from human workers through safety fencing or large distances. Such industrial setups require high degrees of automation with few human operators. Increasingly, there is a trend towards reintroducing human workers to the factory floor to work side-by-side with collaborative robots (Cobots). The coexistence of humans and robots leverages human creativity and general intelligence, as well as robot precision and repeatability and highly increased perception due to rapid developments in artificial intelligence technologies. Emerging avenues in Human-Robot Collaboration (HRC) focus on human centricity, where a symbiotic relationship between humans and robots is envisioned and human performance and perception can be enhanced by using exoskeletons, cognitive ability, co-intelligence, mixed reality, and Brain-Computer Interface (BCI) [10].

There can be an array of possible hazards during HRC stemming from the design of the industrial process, malfunction of the control system of the robot, the robot characteristics (such as speed, force, end-effectors, etc.), or mental stress to the operator during collaboration [11]. The strategies employed to ensure safe human-robot collaboration include limiting tool center point (TCP) velocity, limiting robot power and force, collision detection and avoidance, protective stop functions, and ergonomic design of the robot as well as the working space [12].

### 1.3. Human-Centric Digital Twins

**Figure 1:**
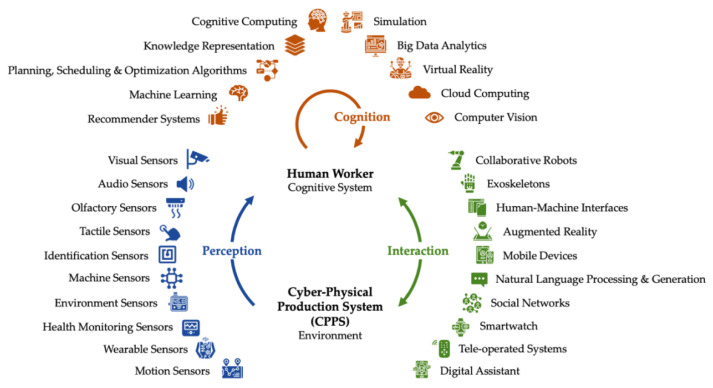
Symbiotic human-machine relationship in the Industry 5.0 [17].

Human centricity is an important hallmark of “Industry 5.0”, which is envisioned to be a value-based industrial revolution with a focus on human well-being, sustainability, flexibility, and efficiency [13]. The challenge of improving human well-being and reducing harmful emissions in smart manufacturing requires structural changes, and may even necessitate transformation to a post-growth economy in high-income countries [14]. This comes with the realization that the objective of automation is not the elimination of labor altogether but to leverage the creativity, objective thinking, dexterity, and decision-making power of humans alongside the repeatability, accuracy, and convenience of using robots for repetitive, labor-intensive, tedious tasks and tasks that may be hazardous for humans [15]. This gives rise to the idea of Operator 5.0 [16], where human perception, cognition, and interaction capabilities are enhanced by a range of enabling technologies, as shown in Figure 1, with the aim of using these strengths to achieve sustainable development with a focus on social well-being and robustness in the face of unexpected challenges.

In order to better integrate humans in CPS, the concept of Human Digital Twins is finding increasing popularity in order to better monitor, evaluate, and optimize human performance, ergonomics, and well-being [18]. The development of Human Digital Twins involves the deployment of a model of humans using sensor data that provides insight into their behavior and attributes, which may include their physical, physiological, cognitive, and emotional states [19]. Although DTs of machines have found broad use in industry, the use of DTs and parallel societies for human-centric social computing is still a developing research topic [20]. Research on human intent recognition is also motivated by the aim of developing symbiotic collaboration between robots and humans so as to distinguish between accidental contact and active collaboration and develop an intuitive and helpful cobot motion control strategy [21]. Creating a symbiotic human-robot collaboration system requires the use of dynamic monitoring of humans and resources using smart sensors, active collision avoidance, dynamic planning, and context-aware adaptive robot control [22].

**Figure 2:**
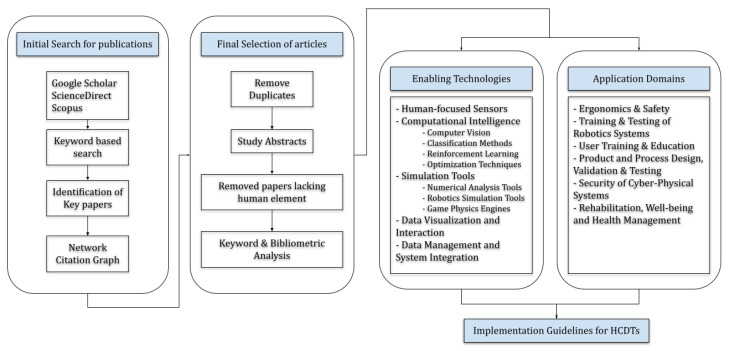
Methodology & Structure.

The main purpose of this study is to present state-of-the-art research conducted on Human-Centric Digital Twins, their enabling technologies, and implementation frameworks for different industrial applications. Firstly, the recent literature in the domain of HCDTs, how it evolved over the years, and areas for future research are discussed using a detailed literature review. Secondly, enabling technologies used by various researchers and engineers in the past and those having potential in the future are discussed. Finally, different applications of HCDT technology along with implementation frameworks are presented, and general guidelines are discussed for the development of HCDTs, as shown in Figure 2.

## 2. Review Methodology

A comprehensive literature review is conducted in this study, where relevant research studies are exported into a digital library, assessed, and screened for any relevance and duplication. The research articles considered in this study were published between the period of 2012 and 2022 and exported from Google Scholar, Science Direct, and Scopus. These databases are selected as they contain the latest full-text peer-reviewed articles and advanced search options and cover the largest content of published research papers. The keywords used to perform a keywords-based search method are as follows: “Digital Twin” AND (“Human-Centric” OR “Human Centered” OR “Human Robot Collaboration” OR “Industry 4.0” OR “Human Robot Interaction” OR “Human Digital Twin” OR “Human-Centered Design” OR “Industry 5.0”).

**Figure 3:**
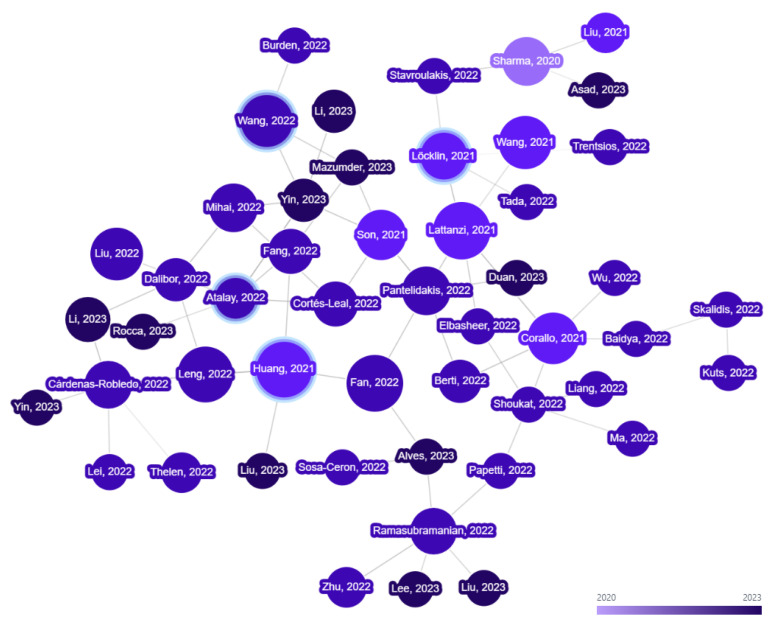
Citation Network Graph.

In the second phase, a graph-based search method is used to find additional relevant papers, where key papers identified from our initial search are used as seeds. For the selection of key papers, the number of citations of the publications was considered, and some of the most highly cited articles included in this review (Table 1) are used as seed papers. Citation analysis tools (inciteful (https://inciteful.xyz/ (accessed on 12 January 2023)) and Litmaps (https://www.litmaps.com/ (accessed on 15 January 2023))) are utilized to create citation network graphs (Figure 3), and related papers are explored and added to the database using the network graph.

Using this search method, first, 237 research publications are screened by studying the abstracts. Relevant papers are imported into the digital library, created using Mendeley software, and further assessed for duplication and redundancies. Only those papers are selected where the human element of DTs is an important consideration in the research work. Finally, the final literature volume of 119 latest publications from 2016 to 2022 is included in this review.

### 2.1. Keyword Mapping and Bibliometric Analysis

**Figure 4:**
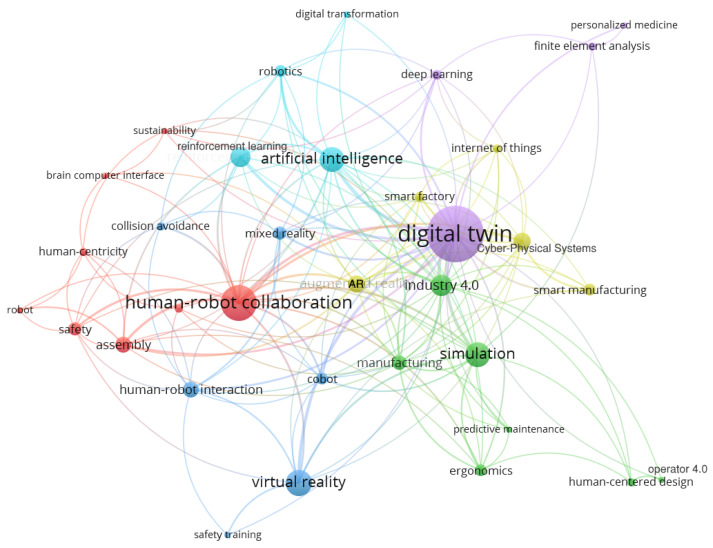
Keyword Co-occurrence Network Map.

A keywords co-occurrence map of selected publications is obtained using bibliometric analysis in VOSViewer [23] and shown in Figure 4. The main keywords are represented by circles and labels; colors represent keyword clusters, and the number of occurrences is shown by circle size. The distance between keywords shows how closely they are linked. The larger distance between two keywords shows that the correlation between the two words is weak, while the smaller distance shows a strong correlation. Figure 4 represents the main keywords in selected literature such as ‘human-robot interaction’, ‘virtual reality’, ‘industry 4.0’, ‘artificial intelligence, and ‘cyber-physical systems’ from different clusters that are closely related to ‘Digital Twin’. The number of occurrences of each keyword and linking strength are also shown in Table 2.

**Figure 5:**
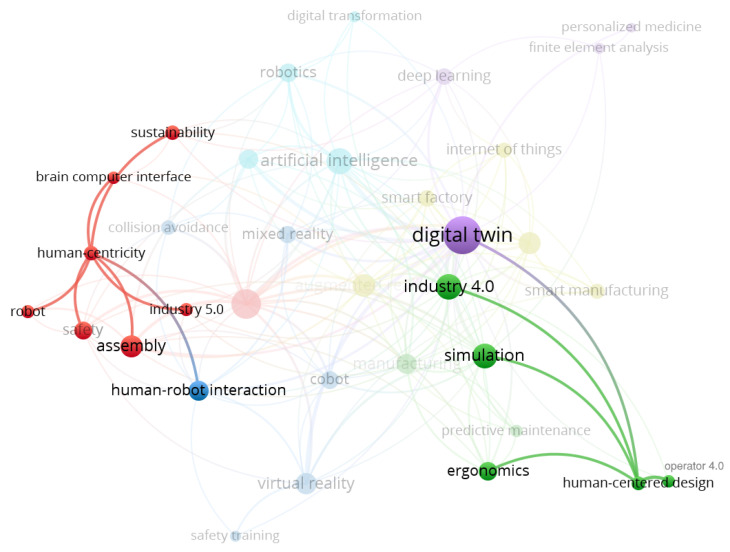
Human-centricity Co-occurrence Network Map.

Figure 5 highlights only the cluster of keywords related to ‘human centricity’ and ‘human centered design’. It shows that selected recent literature has discussed human centricity in DTs, but it is not emphasized in all articles, as Digital Twin literature has not considered human participation in previous years. The same trend is also shown in Table 2, where the keyword ‘digital twin’ has the highest overall link strength of 88 and the keywords ‘human-centricity’ and ‘human-centered design’ have a combined link strength of 12, showing a weak correlation as they occur only six times.

### 2.2. Related Reviews

In the last two years, a number of review articles on DT-related themes have been published, with their focus and outcome summarized in Table 3. Kunz et al. [24] suggested that nearly all papers “neglect the human factor”. Hosamo et al. [23] concluded that Occupant Centric Building Design is “least developed”. A review of Digital-Twin driven smart manufacturing by Lu et al. found that even though DTs for people can increase understanding of well-being and improve working conditions and training programs, over 95% of DTs are developed for manufacturing assets or factories, not for humans [25]. These review papers highlight the need for human-centricity in their respective domains. Based on the literature review, key enabling technologies and application domains for HCDTs are identified and discussed in the subsequent sections.

## 3. Enabling Technologies

A number of technical challenges exist in the implementation of HCDTs. This paper discusses the key technologies that can be used for the development of HCDTs and implemented by different researchers and engineers in the literature for different industrial applications, including Human-Robot Interaction (HRI). The following sections focus on sensing technologies, computational intelligence techniques involving artificial intelligence, optimization and control systems, multiple simulations, and visualization tools. In the end, a generic framework for HCDT is also presented that incorporates the discussed enabling technologies.

### 3.1. Human-Focused Sensors

To create HCDT-driven CPS, sensors can be deployed to collect digital data from humans and other physical systems, which are further integrated with different modeling and simulation tools to support the whole DT framework. Many mature solutions have been developed to collect data from physical systems, such as data collection from Cobots. However, data collection from humans is still in progress. Various human motion tracking sensors developed in recent decades are able to provide accurate results. However, gaze tracking [30], facial temperature, and other unobtrusive and miniaturized psychological and physiological data sensors are continuously evolving, making sensing the mental status of humans still a point of contention [31].

For accurate human skeletal tracking and joint monitoring, optical and non-optical sensing devices were used by researchers. Optical marker-based devices, comprising active and passive markers, such as Optitrack (https://optitrack.com/ (accessed on 12 December 2022)) and VICON (https://www.vicon.com/ (accessed on 12 December 2022)), are widely used for human motion tracking. On the other hand, optical marker-less technology and video-based human motion tracking devices, including RGB-D cameras [32,33], infrared cameras [34,35], and Kinect [36,37,38,39], are also extensively used in literature. Non-optical tracking devices include wearable inertial and magnetic measurement units (IMUs) [40], and magnetometers have been used in the past to track human movements, trajectory, and position while collaborating with cobots. Mechanical motion capture systems are also used when direct measurement of human motion is essential [41].

Different biological sensors are also being used to measure the physiological data of humans to monitor human behavior during human-robot collaboration [42]. Physiological sensors, such as Electrooculogram (EOG) [43], Electrocardiogram (ECG) [44], Electroencephalogram (EEG) [45], Magnetoencephalogram (MEG) [46], and EMG [47], capture signals generated from the human body and can infer important information. Lately, these signals have been broadly used in HRC systems to predict the intention of human operators [46].

Sensors are also required to collect and transmit data on various environmental parameters such as airflow, humidity, light, noise, temperature, and others. This data is then used to create an accurate digital representation of the physical environment, which can be used for simulations, analysis, and decision-making with regard to human comfort and well-being. For example, sensors can be used to monitor the air quality in a building, which can help identify potential health hazards or optimize the operation of HVAC systems in buildings, leading to improved comfort and energy savings.

### 3.2. Computational Intelligence

Artificial intelligence and machine learning are central to realizing the promise of HCDTs. In the context of path and motion planning for robotics systems, model-based control systems are still widely used despite being challenged by data-driven approaches. Model-based control theory can offer advantages such as explainability and performance and safety guarantees [48], which are still lacking in AI-based methods. Developing trust in autonomous systems is an active research area that will be central to achieving symbiotic human-robot collaboration.

#### 3.2.1. Computer Vision

Creating a high-fidelity Human Digital Twin may involve recognition of human facial features, expressions, poses, gestures, and so on. Deep Neural Networks and Computer Vision are being used extensively in literature for this purpose. Yi et al. [32] use RGB-D sensors for posture estimation using CNN. Dimitropoulous et al. [33] also employed an array of technologies to enable an AI system to safely and ergonomically interact with human operators. The AI system uses convolutional neural networks with RGBD sensors and real-life as well as virtually generated imagery as training data to perform object recognition as well as human pose estimation. Machine-learning based computer vision has been widely used in the literature for safe Human-Robot Collaboration [49,50].

#### 3.2.2. Classification Methods

Supervised learning-based classification techniques such as Convolutional Neural Networks (CNNs), Long Short-term Memory (LSTMs), Hidden Markov Model (HMM), and Spiking Neural Networks (SNN) have been used in the literature for motion and intent detection and prediction in HRC [51,52,53]. Yi et al. demonstrated 3D human pose estimation using six inertial sensors and a deep neural network, using data fusion by combining a data-driven and motion-driven approach [40]. Supervised learning is also used to classify EEG signals in Brain-Computer Interface.

The use of BCI in HMI is still in its early stages. Dmytriyev et al. demonstrated the use of BCI based on EEG signals in a collaborative assembly setting, where the operator looks at monitor screens in order to issue commands to the robot controller [54]. Signal classification (unsupervised learning) has also been used in BCI applications; for example, a blink detection algorithm is used by [55] in the context of a collaborative assembly with the aid of an Augmented Reality (AR) device. Machine learning can also be used to improve the capabilities of robot sensors and actuators, which further enhances human-machine interaction. Jin et al. developed a soft-robotic sensory gripper that uses an SVM-based machine learning algorithm for object recognition through tactile feedback [56].

#### 3.2.3. Reinforcement Learning

In Reinforcement Learning (RL) algorithms, agents learn their control policies through unsupervised learning without the need for vast amounts of training data. Deep Reinforcement techniques leverage deep neural networks in combination with RL algorithms such as Q-Learning to solve complex problems and have been shown to surpass human performance in many situations [57]. Reinforcement learning has found widespread use in localization and mapping [58], motion planning [59,60], and in the context of decision-making in human-centric applications [61,62,63] as further described in the applications sections.

#### 3.2.4. Optimization Techniques

Increasingly data-intensive artificial intelligence methods are being used for computational intelligence. However, a number of optimization techniques, such as simulated annealing [64], ant colony optimization [65], Genetic Algorithms [66], have also been used in DT literature, as described in subsequent sections. Kennel-Maushart et al. [67] use Newton’s Method to optimize the solution of the inverse kinematics problem to enhance teleoperation performance via mixed reality for multi-robot systems.

### 3.3. Simulation Tools

For the development of a DT, different simulation tools and packages are required to create an interaction between physical objects and their virtual twins. Many free and commercial simulation environments are available that can be used in the design of DT for HRI, which will be discussed briefly in this section. However, the selection of a specific simulation tool is entirely dependent on the DT application.

#### 3.3.1. Numerical Analysis Tools

Finite Element Analysis (FEA) is often necessary for multiphysics simulations of physical assets involving structural analysis, fluid flow, or thermal loads. Finite Element Analysis tools, such as ANSYS [68,69], COMSOL [66], and ABAQUS [70], have been frequently used to create DTs for biomedical applications and physical assets for which health/condition monitoring is desired [71,72] FEM-based high-fidelity physics simulations are generally computationally costly. Reduced-Order Models based on FEM Simulations can be a useful tool to deploy DTs at scale for complex systems [72], for which commercially available software such as ANSYS Twin Builder (https://www.ansys.com/products/digital-twin/ansys-twin-builder accessed on 14 December 2022) are becoming increasingly popular [73]. As a high-level numerical computing platform, MATLAB is used in a number of studies involving DTs in the medical domain [70,74,75], as further discussed in the applications.

#### 3.3.2. Robotics Simulation Tools

Robotics simulators allow developers and researchers to design, test, and evaluate robotic systems in virtual environments. Gazebo is one of the most commonly used simulators in the context of HCDT literature [60,65,76,77]. It is an open-source simulator that can be integrated with ROS/ROS2. Robot Operating System (ROS) is one of the most widely used platforms used by robotics researchers for a broad domain of applications, including motion planning, control, sensing, localization, and mapping [78]. For robotics manipulation and planning tasks, Gazebo is often used with the MoveIt framework [79], which provides state-of-the-art algorithms for motion planning, collision checking, kinematics, control, and visualization of manipulators. MATLAB/Simulink also provides toolboxes for robotics simulations, such as the Robotics System Toolbox. It can be used in co-simulation settings with other tools such as ROS or game engines such as Unity. Andaluz et al. [80] described a co-simulation scenario involving teleoperation and autonomous control of a robotic arm where Windows Inter-process Communication (IPC) was used to communicate between MATLAB and Unity. Other commonly used robotics simulators include CoppeliaSim [81], and NVIDIA Isaac Sim [82].

#### 3.3.3. Game Physics Engines

Game development applications and physics engines developed for the gaming industry are valuable tools for the development and simulation of DTs [83]. The most commonly used physics engine in the gaming industry is NVIDIA’s PhysX, which is used by Unity and Unreal Engine, the two most popular game development platforms. In recent years, these have widely been used in the development of DTs. For example, Kuts et al. [84] implemented a custom C# script in the Unity game engine to develop a controller for the DT of a Motoman GP8 industrial robot created using Autodesk 3ds Max and Autodesk Maya. Other works that employ game engines are discussed in subsequent sections [30,63,77,85,86,87,88].

### 3.4. Data Visualization and Interaction

Different 3D visualization and rendering software is used by researchers for design visualization as per the requirements of the application area. In immersive user experiences, AR/VR technologies offer new possibilities. The visualization tools and technologies that can be used with DTs are briefly discussed in this section.

#### 3.4.1. Photorealistic Rendering

The creation of virtual models in DTs begins with 3D modeling tools, which can be further used by simulation tools. Some of the tools used in the cited literature include Blender [89], Autodesk 3ds Max [68,90], CATIA [91] and Siemens NX [92]. In addition, the ability of these tools to create photorealistic rendered images can be used to create training data for ML algorithms and to provide a better realistic user experience for the design and inspection of DTs. In many cases, this is done by exporting the models into platforms such as NVIDIA Omniverse [93], Unity [26], and Unreal Engine [86].

#### 3.4.2. Immersive User Experience

The use of DTs creates opportunities to create a rich immersive user experience through, for example, the use of virtual reality, augmented reality, and mixed reality. In augmented reality, virtual objects are superimposed on real images, using a Head Mounted Display (HMD) such as Microsoft Hololens, by using sensors and trackers along with handheld or fixed displays, and AR development tools such as AR Toolkit [94], Microft Mixed Reality Toolkit [24], and PTC Vuforia [92,95]. Mixed Reality (MR) allows for co-existence and interactivity between the physical and the virtual environment [96], which can be used, for example, for intuitive, user-friendly teleoperation of robotic manipulators [97]. In design and manufacturing, AR systems have found widespread applications in training and guiding operators in areas such as operations, maintenance, and quality assurance; however, user acceptance is limited by challenges such as cost, complexity, weight, data security, and privacy issues in AR systems [98]. Interactivity in VR/AR may not be limited to handheld controllers with improvements in gesture tracking through computer vision. For example, Ref. [99] used four monochrome cameras mounted on a VR HMD (Oculus Quest VR) for accurate detection of hand motion, which may be used for a more immersive interactive experience.

Human cognitive ability can be enhanced by employing not just a visual relay of information to the operator through a mixed reality approach as required, but also through haptic feedback and auditory cues and communication. Advances in speech recognition, text-to-speech, and turn-based conversational systems can potentially enhance human-robot interaction. However, there are still major challenges since conversational systems are trained using specific human-human corpora, and their generic practical utility in human-robot interaction remains unproven [100]. With increasingly sophisticated Large Language Models (LLMs) such as Open-AI’s ChatGPT, it becomes foreseeable for rich impromptu human-machine interaction in industrial settings to be carried out via two-way text and voice communication through a fusion of technologies such as Task and Motion Planning, Vision, Language, and Control in Robotics [101].

### 3.5. Data Management and System Integration

In a Human-Centric Digital Twin, there is interconnectivity between humans, robots (or miscellaneous machines), the environment, and their DTs in the physical and virtual domains. In the implementation of Digital Twins, a wide range of tools and technologies for data management, including data transmission from devices and sensors, data storage, and data fusion, have been used. Industrial IoT platforms such as Siemens MindSphere and GE Predix are gaining popularity for system integration in DTs [102], especially for conventional infrastructure management. In Human-Centric Digital Twins, the cited literature uses more research-oriented tools and standards, such as ROSBridge [79,86,94], MQTT [32], and MTConnect [87] for communication. ROSBridge is a WebSocket server that allows web browsers to talk to ROS. MQTT (Message Queuing Telemetry Transport) is a lightweight machine-to-machine messaging protocol. MTConnect is an open-source standard that provides a semantic vocabulary for manufacturing equipment. System integration to ensure a seamless, secure flow of data to represent physical infrastructure, especially for legacy systems, remains an important challenge in the deployment of DTs [28].

**Figure 6:**
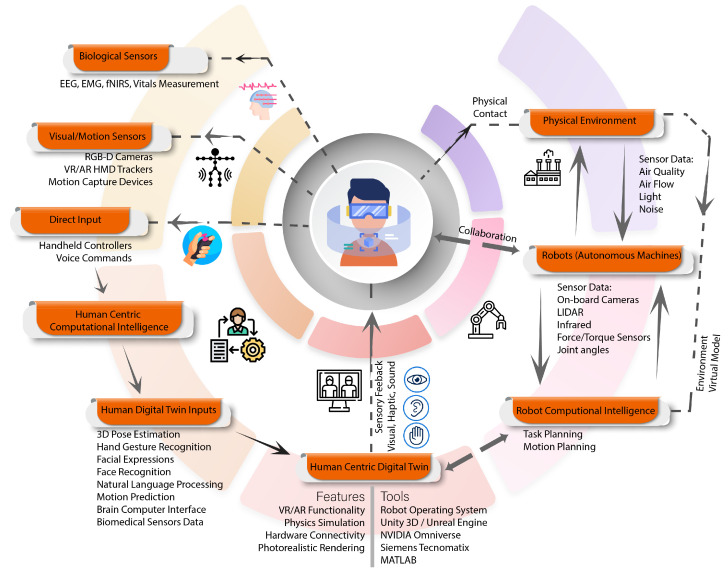
Enabling Technologies and framework of Human-Centric Digital Twins in Industry 5.0.

A generic framework for HCDTs is presented in Figure 6, in which many of the devices and methods that may be employed are listed. A virtual model of the environment can be created using CAD models or by reconstruction using image-based or 3D point-based modeling approaches [103]. The design and selection of sensors, computational tools, simulation tools, data visualization tools, and data management tools in the integrated system are carried out according to the specific requirements of the HCDT based on the application area.

## 4. Application Domains

Digital Twin Technology has recently been applied in a range of industries and scenarios, including Smart City construction [104], monitoring and optimization of physical assets including machine tools, vehicles, machinery, mechanical structures, and materials [27,28,29]. Here, we have identified and placed particular emphasis on key application domains where human-centricity is of paramount importance.

### 4.1. Ergonomics and Safety

In Smart Factories, as humans and collaborative robots begin to share workspaces without borders, it is necessary to ensure human safety and optimize ergonomics during such interactions. Cobots are designed to be intrinsically safe due to speed, force, and torque limits and design considerations. Additionally, there is extensive literature, guidelines, and standards dealing with risk assessment and safety requirements for human-robot interaction [105]. Havard et al. [106] created a DT involving the co-simulation of a CPPS using Dassault Digital Factory Suite and Unity 3D for an assembly operation using a UR10 cobot. The Digital Twin was employed to carry out a safety and ergonomics assessment in VR. Agnusdei et al. [29] provide a framework to assess the capability of a DT to improve safety based on three criteria. The first relates to data acquisition, where real-time data acquisition is heavily favored. The second criterion relates to data processing. DTs use statistical methods, multi-physics modeling, or artificial intelligence-based methods for data processing. And the third criterion is about the source of risk, which can be the machine, the human, or human-machine interaction.

In HRC safety, collision avoidance is an important challenge, commonly addressed in literature [107]. A range of techniques are employed in literature where DTs are employed for safe contactless interaction with human operators. In ref. [36], an Oculus Rift HMD and Kinect sensor are used by a collision avoidance control algorithm. Liu et al. [37] employ a novel deep reinforcement learning algorithm, IRDDPG, to allow a Cobot to reach a target state while minimizing the risk of collision with a human hand, modeled as a bounding box using a Kinect V2 depth camera. In numerous studies, DTs have been leveraged to optimize ergonomics in a manufacturing setting. Greco et al. used a DT simulation in Siemens Tecnomatix Jack and optical motion capture using Microsoft Kinect (R) and an indigenously developed wearable motion capture system to monitor and optimize the ergonomics during a manufacturing task by evaluating and minimizing operational health and safety-related risk factor metrics [38].

**Figure 7:**
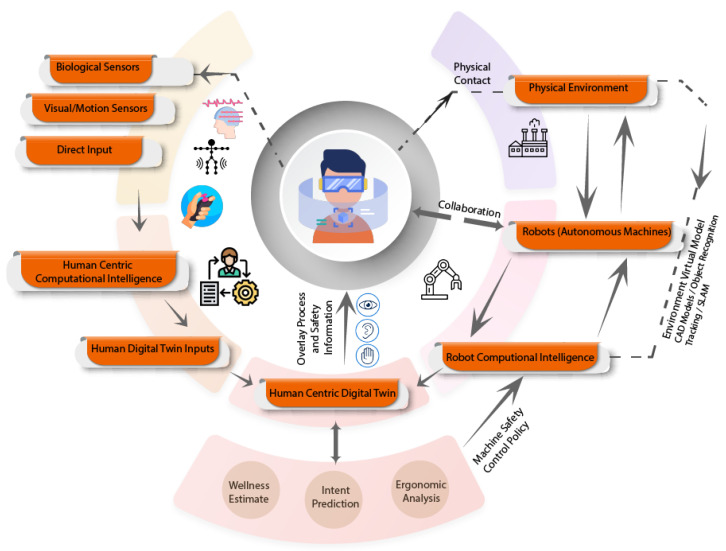
Generic framework for HCDT for safety and ergonomics.

Choi et al. [39] created a DT of an HRC scenario with a human operator and a UR3 cobot using DL algorithms applied to 3D point cloud data from two Azure Kinect depth sensors and conveyed Safety and Task information to the human operator using a Mixed Reality HMD (Microsoft HoloLens 2). Table 4 presents a brief summary of related literature. A generic layout for HCDTs for ergonomics and safety is shown in Figure 7. The framework aims to enhance human well-being and safety by monitoring biological sensor data and creating a human intent model, which can be used by machine intelligence to optimize ergonomics and safety.

### 4.2. Training and Testing of Robotics Systems

**Figure 8:**
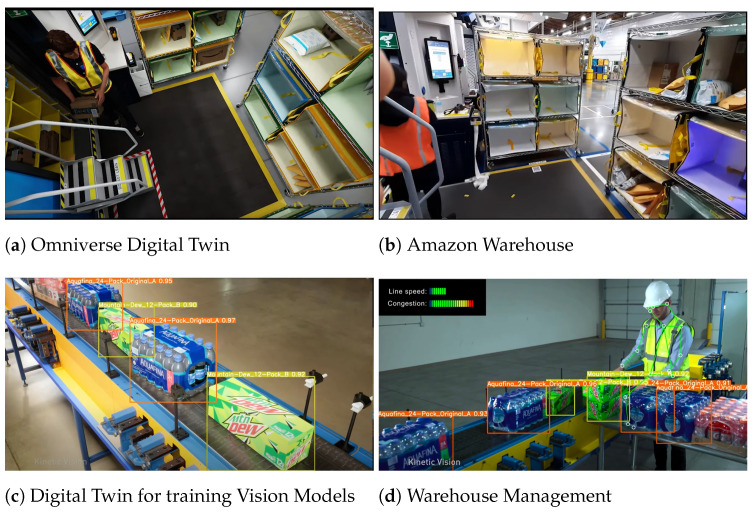
Nvidia Omniverse based DTs for Amazon and Pepsi [111].

Digital Twins have found a lot of utility in the training and testing of robotics systems. Training supervised machine learning algorithms is highly data intensive. DTs can provide a high-fidelity virtual environment to generate a vast amount of test data to viably train an ML model and transfer it to the physical system/robot. NVIDIA has demonstrated this capability using its newly launched platform, NVIDIA Omniverse, with industrial partners such as Pepsi and Amazon to create DTs of warehouses and distribution centers for training AI models [111], as shown in Figure 8.

Mania et al. employed photorealistic simulations rendered in a game engine (Unreal Engine 4) to increase the performance of the perception system of a mobile robot by comparing its detection results with an expected result that is acquired through physics simulations in the virtual environment [86]. Table 5 presents a brief summary of related literature.

**Figure 9:**
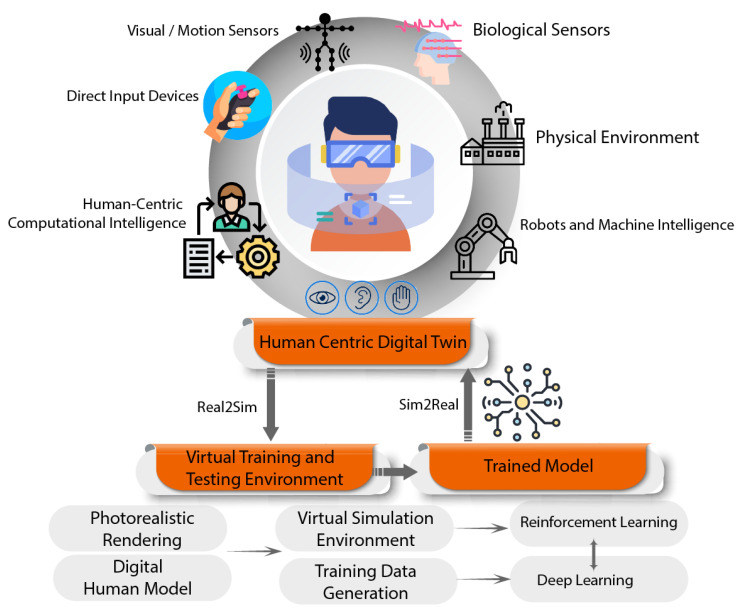
Generic framework for Robotics System Training.

A generic framework for using HCDTs for training and testing of robotics systems is shown in Figure 9. Here, the intent is to realize the aim of a symbiotic human-robot relationship, by training a deep learning or deep reinforcement learning algorithm using human and machine data fed into the DT, which includes predicting human intent and creating a machine policy to assist the human in a flexible intuitive collaborative environment.

### 4.3. User Training and Education

VR/AR technologies are already widely used in training and teaching [113] in educational, industrial, and military applications. The use of AR/VR headsets enables operators in process industries to get a better understanding of the process, for remote assistance and operator training [28]. Um et al. showed that the distribution of computing power allows for image recognition or various detection algorithms to be used seamlessly with Microsoft Hololens to guide the operator in a production setting. DTs have also been used in enhanced surgical training, where the use of DTs with haptic feedback is reported to increase accuracy and reduce cognitive load on surgeons [114].

In light of the COVID-19 pandemic and subsequent travel restrictions, there is increased recognition of the utility of AR as a distance learning and remote assistance tool in education and industry. For example, a case study [115] from PTC Vuforia highlighted how local field engineers from Rockwell Automation used AR as a remote assistance tool to get virtual support from senior engineers who could not be present due to travel restrictions for the installation of specialized equipment. In VR-based training, the use of artificial intelligence and optimization methods has shown to be effective tools for adaptive, customized training based on the user’s cognitive and physiological abilities [116]. Unity and C# were used by ref. [117] in a VR-based training with turn-based dialog for adaptive de-escalation training.

In ref. [94], an AR Headset (Microsoft HoloLens) is used to receive input from ROS through the Rosbridge communication package to enhance the user’s experience and perception. VR/AR-based training with DTs is receiving a lot of interest in a wide range of industries, such as construction [118,119], mining [120]. Using immersive virtual simulations, the safety perception of HRC in construction workers is tested and enhanced in [121]. Matsas et al. [122] created a virtual training and testing environment using 3D graphics generated in 3ds Max in a gaming engine (Unity 3D). Using a VR HMD, user evaluations of safety techniques adopted by the HRC AI are carried out and analyzed. Wang et al. [35] used a combination of an industrial camera, a VR HMD, and Unity for the assessment of Welder behavior in a teleoperation setting with a UR5 Cobot.

In the context of user training and education, the literature shows that immersive technologies, audio-visual feedback, and the use of artificial intelligence with Digital Twin technology are central to achieving adaptive, intuitive, and customized user assistance and training experiences.

### 4.4. Product and Process Design, Validation and Testing

**Figure 10:**
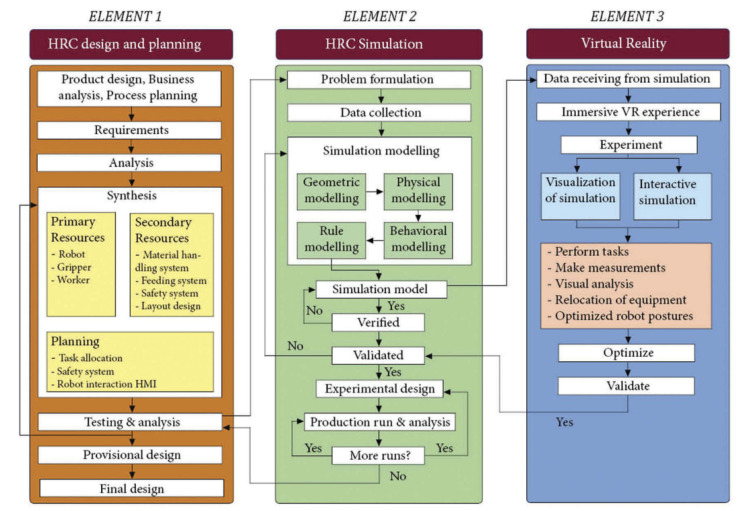
VR enhanced Digital Twin framework for HRC design [123].

The prospect of using DTs in combination with immersive technologies such as virtual reality and augmented reality (VR/AR) can be utilized to enhance and enrich the process and product design in manufacturing settings. Malik et al. [123] leveraged DTs and virtual reality for HRC design and planning. The proposed architecture uses bi-directional information sharing between the physical and virtual robots and a human operator using TCP/IP communication, and motion capture technology. In the VR environment, assisted by a virtual assistant (chatbot), designers will have the ability to manipulate objects and study the HRC design from the perspective of visibility, reach, and ergonomics to optimize cycle times and safety. The framework proposed by the authors is shown in Figure 10.

Kousi et al. [79] employed ROS Gazebo, MoveIt, and Lanner Witness Simulation in an automotive assembly case study, where a DT-based system was used to generate alternative configurations for the assembly process with a dual-arm mobile robot and human operators and validate the system’s performance. Wang et al. present a framework for Human-Robot Collaborative Assembly using DTs that proposes a data fusion and visualization service to process information coming from human-centric as well as robot sensors [124]. The data is subsequently processed and used to generate events, schedule tasks, and run the robot’s control service. Table 6 presents a brief summary of related literature.

The literature shows that DTs have been leveraged within this application domain to optimize cycle time, enhance workstation layout, ergonomics, and flexibility, and carry out dynamic scheduling, line balancing, and process planning.

### 4.5. Security of Cyber-Physical Systems

**Figure 11:**
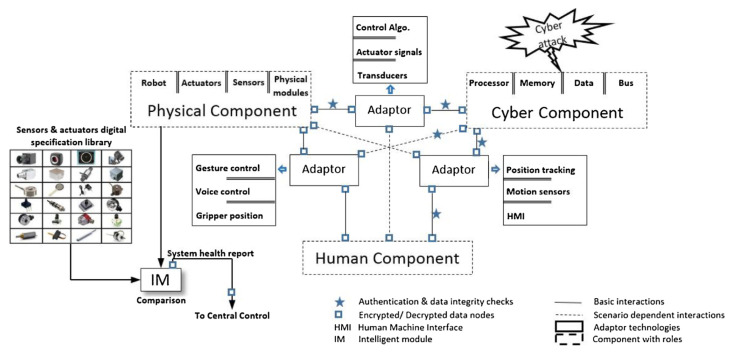
Framework for Human-Robot CPS under Cyber-attack [135].

Security and reliability are important issues in DTs, more so in HCDTs. Humans may be involved in the planning and execution of the attack and may also be the target of an anthropocentric CPS (ACPS). In the context of a Collaborative Robotic Cyber-Physical System (CRCPS), Khalid et al. present an assessment of attack types, possible effects, and severity and present a framework, shown in Figure 11, for a secure CRCPS based on authentication and data integrity checks and an independent module to compare real-time sensor data to a pre-stored specifications library and report any discrepancies [135].

Security becomes even more central in mission-critical applications such as robotic surgery. Laaki et al. investigate the challenges of latency and security in teleoperated robotic surgery over mobile networks [136]. The authors used an HTC Vive HMD with a haptic feedback controller and a DT created in Unity3D with a 4G mobile connection routed through a VPN server, secured via password protection and biometric authentication, and highlighted security issues such as protection of intellectual property rights, protection of data, and denial-of-service attacks.

Digital Twins and their enabling technologies can themselves be used for enhancing security. Deitz et al. set out the formal requirements of a security framework that employs DT simulations to detect, analyze, and handle security incidents [137]. Blockchain technology can be utilized to ensure the reliability and security of Digital Twin data for CPS [138]. A brief description of some other works in this area is shown in Table 7.

### 4.6. Rehabilitation, Well-Being and Health Management

In the medical domain, Human Digital Twin technology has found more maturity and acceptance. Medical Digital Twins (MDTs) have been used in many areas, including pulmonology [140], orthopedics [91], and hepatology [75]. In a recently publicized campaign, the National Football League (NFL), in partnership with Amazon AWS is working towards creating a ’Digital Athlete’ with the aim of improving player health and safety [141]. This may further increase public interest in MDTs.

MDTs may involve using clinical imaging techniques to develop patient-specific solutions, for example for the human tongue [73], tibia [69], and aortic walls [70]. Table 8 presents a brief description of these and other related works.

The use of DTs in medicine is not without challenges or apprehensions. Data-driven patient-specific medical care can potentially lead to segmentation and discrimination, with an increased need to ensure privacy and transparency in data usage [142].

**Figure 12:**
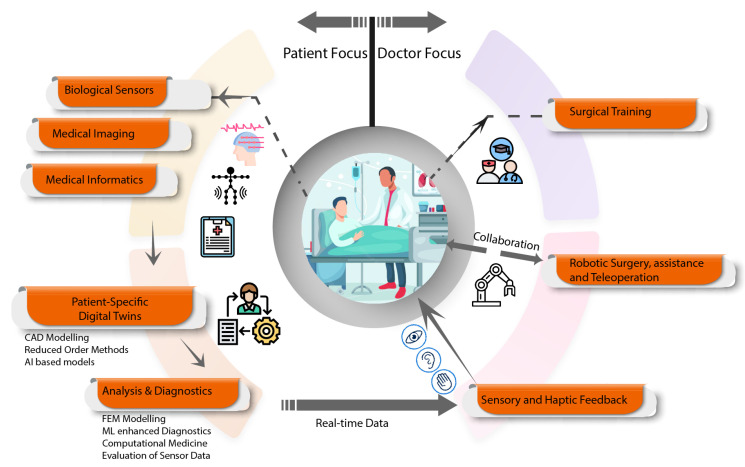
Generic framework for DTs in healthcare.

Figure 12 illustrates a generic layout for healthcare applications. Patient-specific DTs are generated using information from sources such as medical imaging, biological sensor data, and medical informatics. Analysis and dagnostic data based on the DT is relayed to healthcare professionals, where DT-enabling technologies may aid healthcare professionals in training and enhancing robotic surgery through sensory and haptic feedback.

## 5. Discussion

Cyber-physical systems with synergistic human-machine interaction, aided by Human-Centric Digital Twins are slowly making their mark in multiple application domains. Regarding the human element in Digital Twin applications, the utility of different sensors, Human Digital Twin inputs, feedback mechanisms, and prevalent types of machine learning algorithms in the identified application domains are shown in Table 9. The utility is ranked as low, medium, or high and displayed graphically.

As shown in the table, in order to optimize ergonomics and safety in industrial settings, biological and visual sensor data is often used for pose and motion recognition by adopting deep learning and relating safety-critical information back to the human operator. Human supervisory control and intervention are necessary to ensure real-time safety during operation or to assess and optimize different offline scenarios. For training and testing robotic systems, Sim2Real technology can include the human element by generating virtual training data for deep learning models and testing agent policies using reinforcement learning (RL) techniques in an environment with a digital human created with the aid of human sensor data, for example using visual and motion sensors. This can additionally be aided by human intervention through the use of behavior cloning (BC) methods in RL.

The use of HCDTs with VR/AR may be leveraged to create a personalized and adaptive user learning and collaborative experience. The audio-visual interface and direct input devices play a central role in this application, which may be aided by artificial intelligence by leveraging Large Language Models (LLM) or for adaptive learning. The supervisors of the training regime should have access to intervene as required. Visual and motion sensors are central to process design applications. HCDTs, using optimization techniques and deep learning, can optimize cycle time, enhance workstation layout, ergonomics, and flexibility, and carry out dynamic scheduling, line balancing, and process planning. An immersive visual interface can be very beneficial for human designers to interact with the DT for process monitoring and assessment.

In security applications, biometric and facial recognition, motion sensing, and the use of data security techniques, including blockchain technology, are central. Here, deep learning techniques can be leveraged to provide safeguards against the numerous types of cyberattacks. Human intervention is required only in the event of any detected security breach or threat. HCDTs already have a lot of acceptance in medical applications, where DTs of patients are created using medical imaging and biological sensors, which can be subjected to deep learning algorithms or FEA analysis. These are used by doctors who can train and operate with the aid of immersive sensory feedback, including haptic feedback and robotic assistance.

## 6. Conclusions

In this research work, a state-of-the-art literature review on Human-Centric Digital Twins (HCDTs) and their enabling technologies is conducted. A key shortcoming observed in current DT literature is that the focus is almost entirely on the physical assets of a CPS and not on human operators. In the coming years, an increasingly ambitious and sophisticated industry and process-specific DTs may address this shortcoming by developing HCDTs. A generic framework is proposed to underline the enabling technologies, such as human-focused sensors and computer vision, that can be used for the creation of HCDTs. The enabling technologies for this purpose have received considerable individual attention. For example, AI-driven algorithms for a range of problems such as object recognition and collision avoidance or using DTs for monitoring and supervision of physical assets, etc., have reached some degree of maturity. However, there is a lack of literature on how all these enabling technologies are utilized together in a synergistic manner to enhance human-machine interaction in industrial settings.

We identified six key application areas for DTs with strong human involvement, which are ergonomics and safety, training and testing of robotics systems, user training and education, product and process design, validation and testing, security of cyber-physical systems, and finally rehabilitation, well-being, and health management. Implementation frameworks for the selected domains highlight the workflow and desired outcomes (such as optimization of ergonomics, security policy, task allocation, etc.). It has been found that the use of HCDTs in literature on the security of CPS is currently underdeveloped and merits further exploration. DTs are being extensively used to train robotic systems by simulation and data generation in a virtual environment. These can be further enriched by including more human-focused sensor data to enable synergistic, intuitive collaboration. The development of increasingly specialized modeling tools and the ubiquitous spread of artificial intelligence can transform this expert-driven paradigm towards becoming user-driven. Over time, the use of HCDTs is expected to considerably expand owing to the immense interest shown by researchers and industry.

## Figures and Tables

**Table 1:** Most cited articles in this review.

S#	Citations	Authors	Title	Year	Journal
1	158	Lihui Wang, Robert X. Gao, József Váncza, Jörg Krüger, Xi Vincent Wang, Sotiris Makris, George Chryssolouris	Symbiotic human-robot collaborative assembly	2019	CIRP Annals
2	155	Kai Ding, Felix T. S. Chan, Zhang Xudong, Guanghui Zhou, Fuqiang Zhang	Defining a Digital Twin-based Cyber-Physical Production System for autonomous manufacturing in smart shop floors	2019	International Journal of Production Research
3	144	S. Nahavandi	Industry 5.0—A Human-Centric Solution	2019	Sustainability
4	130	Koenraad Bruynseels, Filippo Santoni de Sio, Jeroen van den Hoven	Digital Twins in Health Care: Ethical Implications of an Emerging Engineering Paradigm	2018	Frontiers in Genetics
5	82	Nikolaos Nikolakis, Kosmas Alexopoulos, Evangelos Xanthakis, George Chryssolouris	The digital twin implementation for linking the virtual representation of human-based production tasks to their physical counterpart in the factory-floor	2019	International Journal of Computer Integrated Manufacturing
6	74	P. Aivaliotis, Konstantinos Georgoulias, George Chryssolouris	The use of Digital Twin for predictive maintenance in manufacturing	2019	International Journal of Computer Integrated Manufacturing
7	74	Ali Ahmad Malik, Arne Bilberg	Digital twins of human robot collaboration in a production setting	2018	Procedia Manufacturing
8	73	Azfar Khalid, Pierre T. Kirisci, Zeashan Hameed Khan, Zied Ghrairi, Klaus-Dieter Thoben, Jürgen Pannek	Security framework for industrial collaborative robotic cyber-physical systems	2018	Computers in Industry

**Table 2:** Keywords.

id	Keyword	Occurrences	Link Strength
1	digital twin	49	89
2	human-robot collaboration	25	52
3	artificial intelligence	15	37
4	industry 4.0	12	31
5	simulation	15	28
6	augmented reality	9	27
7	cyber-physical system	9	24
8	virtual reality	16	23
9	assembly	8	20
10	manufacturing	7	19
11	reinforcement learning	11	18
12	human-robot interaction	8	16
13	robotics	5	16
14	cobot	5	14
15	ergonomics	5	13
16	mixed reality	6	13
17	deep learning	4	12
18	safety	6	12
19	smart factory	4	12
20	smart manufacturing	5	10
21	internet of things	3	9
22	collision avoidance	3	8
23	human-centricity	3	7
24	sustainability	2	7
25	human-centered design	3	6
26	predictive maintenance	2	6
27	industry 5.0	3	5
28	robot	2	5
29	brain-computer interface	2	4
30	digital transformation	2	4
31	finite element analysis	3	4
32	operator 4.0	2	4
33	safety training	2	4
34	personalized medicine	2	3

**Table 3:** Summary of previously published Review Papers on Digital Twins.

Ref.	Year	Focus	Outcome
[26]	2021	VR/AR solutions in HRC	Challenges for AR/VR solutions are identified, especially with respect to calibration and tracking of objects
[27]	2022	DTs in Manufacturing, classified by publication type, year, country, manufacturing sector	Classified literature by simulation method, and attributes of the physical and digital layers (such as optimization, monitoring, control, etc.)
[23]	2022	DTs in Construction and Facility Management	Building Information Management (BIM) is a more developed and applied concept in construction. Research on the integration of BIM and IoT in a DT framework is further behind
[24]	2022	Augmented Reality in Digital Twins	Identified that AR is used for information visualization, guidance and control in DTs, mostly in areas of Manufacturing, Training, and Construction
[28]	2022	DTs in Process Industry focusing on challenges and barriers in adoptions, as well as enablers	System integration challenges, data, and IP security, performance issues in real-time data exchange and organizational issues, are highlighted as major barriers
[29]	2021	Classification of DT literature in safety domain	A framework for assessment of the capability of DTs to improve safety.
[19]	2022	Defining Human Digital Twins (HDTs), identifying their attributes and use cases	Attributes of HDTs include physical, physiological, perceptual, cognitive and emotional attributes. HDTs are commonly used in health industry and product design and validation.

**Table 4:** Applications in Ergonomics and Safety.

Ref.	Year	Tools	Proposed Idea
[108]	2016	Custom GUI	A custom software, “Human-Industrial-Robot-Interaction-Tool” HIRIT was developed for safety evaluation on HRC, using depth sensors for human motion capture and Genetic Algorithm for safety distance estimate
[32]	2022	CNN Motion Capture	Human pose estimation using RGBD Camera is used for human localization and collision avoidance in HRC
[85]	2021	OpenSim Unity	Ergonomic analysis of pick-and-place task using motion capture, biological sensors and a musculoskeletal human model
[109]	2021	ROS	Ergonomic Analysis and dynamic scheduling in an HRC scenario with a personalized Human Digital Twin with motion analysis and a skin surface
[76]	2022	ROS Gazebo Open Dynamics Engine	Implementing an Actor-Critic based Reinforcement Learning algorithm to ensure collision-free HRC
[110]	2022	Siemens Jack Unity	Ergonomic human-centered design of a tractor dashboard using VR simulations in Unity, ergonomics analysis in Siemens Jack, incorporating analysis of human motion and physiological sensor data

**Table 5:** Applications in Training of Robotics systems.

Ref.	Year	Tools	Proposed Idea
[89]	2021	CNN CAD/Blender	Learning to recognize the orientation of arbitrarily placed plastic parts using CNN based ML model trained via a DT generated training set training
[86]	2020	ROS Unreal Engine 4	Improving Robot Perception by comparing real visual sensor data to expected data generated by photorealistic virtual model
[112]	2022	MATLAB	Using a DT for CNN, pose estimation, and transfer to Yumi Cobot for imitation and teleoperation
[65]	2019	Python ROS Gazebo	Using Ant Colony Optimization to train a robot to reach target states while avoiding obstacles, validated in the DT before transferring to real robot
[87]	2022	Unity ROS	VR based control and robot programming Cobot for a pick and place task and its DT using ROS, Unity, and MTConnect.
[88]	2022	Unity HoloLens	Ad-hoc Robot Navigation and safety visualization in HRC using AR, hand gesture control and an Android application
[77]	2021	ROS, Gazebo, MoveIt, Unity	VR based ad-hoc DT-enabled cobot control in pick and place tasks validated by a user study
[30]	2021	Unity HoloLens	In a DT-enable HRC scenario, using DL (Retina-Net) for object recognition; eye tracking and hand gestures, voice commands to control the system and a DT for visualization through HoloLens
[62]	2021	Siemens Tecnomatix	Used a DT of a PLC controlled Cobot for Deep Q-Networks (DQN) training to develop the capability of dynamic robust scheduling in a manufacturing setting
[63]	2021	Unity TensorFlow	Reinforcement Learning training using TensorFlow utilizing a DT of Robotic arm for pick and place task
[59]	2022	V-REP ROS	Using Deep Reinforcement Learning to train robot policy using DT for grasping operations in the context of assembly
[60]	2021	ROS Gazebo	An architecture for reinforcement training in ROS on a DT, followed by transfer to real is discussed with the help of case studies on a Fanuc Industrial Robot and fleet management of mobile robots
[95]	2022	Vuforia Unity	Implementing multi-robot collaborative teleoperation using AR and DT, using Reinforcement Learning for robot motion planning.

**Table 6:** Applications in Design, Validation, and Testing.

Ref.	Year	Tools	Proposed Idea
[125]	2018	Siemens Tecnomatix	Using a DT in an HRC assembly scenario to optimize workstation layout by analyzing collision, placement, human reach and vision in the virtual environment
[126]	2019	Camunda BPM, Java, XML3D	In a factory floor setting, analysis of human motion and action categorization (such as walking, picking, screwing); subsequent transfer to virtual environment for process optimization, and cycle time and ergonomics evaluation
[127]	2021	Siemens Tecnomatix	Tested, analyzed and optimized a collaborative assembly case study for safety, cycle time, and productivity using a DT with a UR5 cobot
[128]	2019	Unity HoloLens	Using AR and DT in CNC machining operations for visualization and communication (voice/gesture commands)
[66]	2022	COMSOL Multiphysics	DT of an Optical Fiber Drawing Process is used for real-time monitoring and evaluation (using DL algorithms) of process parameters and quality control
[129]	2019	Siemens Tecnomatix	Process planning and configuration for manufacturing of an impeller using industrial robots, machine tools, and an AGV in a with a DT based Cyber-Physical Production System (CPPS)
[130]	2021	VINCENT Unity	Creating a DT of a proANT 436 AGV visualized in a projection system using Unity for material flow simulation in a virtual logistic system
[64]	2022	GAMS	HRC Assembly Line Balancing Problem (ALBP) is simulated using General Algebraic Modeling System (GAMS) Software, Simulated Annealing and Mixed-Integer linear programming (MILP)
[90]	2019	Unity, Visual C#, Autodesk 3ds Max	Implementing a DT of an Intelligent Workshop created using 3ds Max and Unity with real-time synchronization for monitoring and digitization
[131]	2021	AI, Deep-Q Network, GA	Implementation of a reinforcement learning based scheduler for dynamic production scheduling in a smart factory with real machine and operation sensor data
[61]	2022	Openpose DDPG	Using Deep Reinforcement Learning (DDPG) for motion planning with Openpose and Semantic Segmentation for human intent and task prediction in a collaborative assembly-commissioning with an automotive generator as a case study
[132]	2021	D-DDPG	Presented a DT-based HRC assembly framework integrating all kinds of data from digital twin spaces. Double deep deterministic policy gradient (D-DDPG) is applied to optimize HRC strategy and action sequence
[133]	2022	Siemens Tecnomatix, CAM NX	Human-centered design of DT-assisted collaborative shoe polishing, simulated on Tecnomatix, using a novel polishing tool controlled by CAM NX on a UR5 robot
[134]	2021	Java	Implementing a DT-driven smart manufacturing workshop to optimize mass customization for increased flexibility

**Table 7:** Applications in Security of Cyber-Physical systems.

Ref.	Year	Proposed Idea
[137]	2020	Sets out formal requirements of a security framework that employs DT-based simulations to detect, analyze, and handle security incidents
[138]	2022	Demonstrates the use of blockchain technology to ensure reliability and security of DT data for CPS
[139]	2022	A parallel metaverse is envisioned that leverages Blockchain technology to ensure data reliability.

**Table 8:** Applications in Rehab, Well-being and Health Management.

Ref.	Year	Tools	Proposed Idea
[140]	2021	CNN, Oculus MR	MR based Surgery training and analysis for Video-Assisted Thoracoscopic Surgery (VATS) using patient CT image for mesh generation, visual rendering, and haptic feedback
[91]	2021	CATIA, PCA	Using patient-specific DTs developed via CT scans by comparing the real ankle of patients to the DT via machine Learning, the tibiotalar joint axis is identified
[75]	2019	MATLAB, ROM	Patient Specific computational model of human liver anatomy was created using Reduced Order Modelling and Statistical Shape Analysis, solved using Sparse Subspace Learning coupled with an FE Solver
[73]	2021	ANSYS Twin Builder	Creating patient-specific FE model of human tongue, and its ML-based reduced order model for simulate nonlinear behavior of tongue.
[69]	2021	3D Slicer, Simpleware, ANSYS	Patient-specific FE model of tibial fracture based on 3D X-Ray imagery to assess stress distribution and fracture risk under different possible interventions
[70]	2018	MATLAB, TensorFlow, ABAQUS	Stress distribution on aortic walls using Neural Network trained using FEM, where Aorta shape encoding uses PCA applied on real patient geometries
[68]	2023	ANSYS 3ds Max	Quasi-static and dynamic analysis for biomechanical simulation of accidental scenarios during HRC using FEM
[74]	2020	MATLAB	Used machine learning classifiers and statistical tools to model athlete performance

**Table 9:** Guidelines for enabling technologies in application domains.

	Legend:	High Utility:	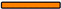	Medium Utility:	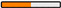	Low Utility:	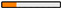
		**Ergonomics & Safety**	**Robotics Training**	**User Training**	**Design & Validation**	**Security of CPS**	**Health & Well-Being**
**Sensors**	Biological Sensors	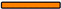	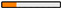	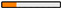	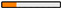	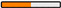	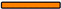
	Visual & Motion	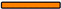	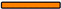	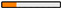	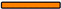	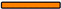	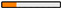
	Direct Input	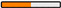	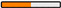	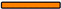	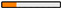	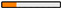	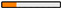
**HDT Inputs**	Pose	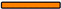	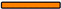	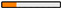	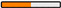	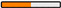	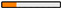
	Hand Gesture	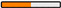	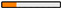	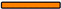	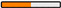	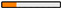	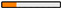
	Facial Expression	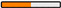	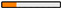	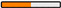	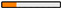	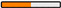	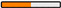
	Gaze	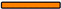	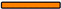	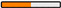	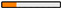	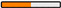	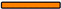
	Motion Prediction	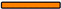	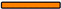	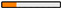	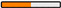	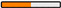	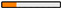
	Language (NLP)	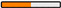	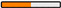	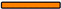	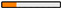	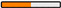	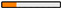
	BCI	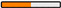	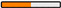	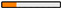	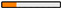	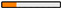	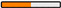
** Machine Learning Requirements**	Optimization	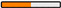	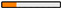	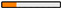	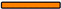	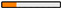	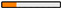
	Deep Learning	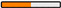	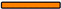	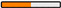	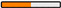	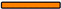	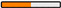
	Reinforcement Learning	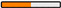	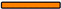	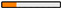	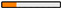	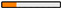	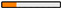
**Feedback and Interface**	Audio	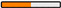	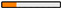	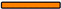	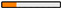	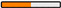	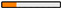
	Visual	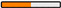	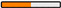	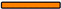	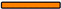	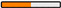	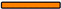
	Haptic	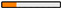	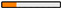	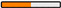	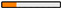	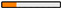	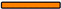

## Data Availability

Not applicable.
